# Associations between tattooed body surface area and maladaptive personality traits in a community sample

**DOI:** 10.1038/s41598-026-42987-x

**Published:** 2026-03-08

**Authors:** Marios N. Adonis, Mark J. M. Sullman, Aigli Athanasiadou, Timo J. Lajunen

**Affiliations:** 1 Department of Social Sciences, School of Humanities and Social Sciences, University of Nicosia, 2417 Nicosia, Cyprus; 2 Department of Psychology, Norwegian University of Science and Technology, Trondheim, Norway; 3 Department of Psychology, University of Helsinki, Helsinki, Finland

**Keywords:** Dysfunctional personality traits, DSM-5 alternative model of personality disorders, Tattoos, Tattoo body coverage, Body modification, Health care, Psychology, Psychology

## Abstract

Evidence for personality differences between tattooed and non-tattooed adults is mixed. Assessing dimensional maladaptive traits may help clarify these associations. A community sample of adults in Cyprus (*N* = 280; *M* = 28.0, SD = 9.5; range 18 to 64) completed the Personality Inventory for DSM-5 Brief Form Adult (PID-5-BF) and a Tattoo Coverage Tool estimating the percentage of body surface area tattooed (tBSA). Analyses included t-tests, correlations, and hierarchical regressions controlling for age, sex, and socioeconomic status. More than half (58.6%) of the participants reported having at least one tattoo. Compared with non-tattooed participants, tattooed participants scored higher on Disinhibition (*d* = 0.47, *p* < .01) and on the PID-5-BF total (*d* = 0.29, *p* = .02). tBSA correlated with Antagonism (*r* = .26, *p* < .01), Disinhibition (*r* = .21, *p* < .01), and the PID-5-BF total (*r* = .16, *p* = .01). Tattoo count showed weaker associations (Disinhibition *r* = .14, *p* < .05; Psychoticism *r* = .12, *p* < .05). In regressions, Antagonism alone explained 6.8% of the variance in tBSA (*p* < .001) and Disinhibition alone explained 4.6% (*p* < .001). In the joint model, Antagonism and Disinhibition explained 7.7% of the variance in tBSA (*p* < .001), with Antagonism emerging as a significant predictor (*p* < .01). Men reported higher tBSA and higher Antagonism, Detachment, Disinhibition, Psychoticism, and PID-5-BF total scores; women scored higher on Negative Affectivity. Tattoo presence was associated with higher Disinhibition and a higher overall maladaptive trait load, while Antagonism best tracked tBSA. tBSA was more sensitive than counts for detecting trait associations. Findings support AMPD-aligned assessment and the use of tBSA in community research on tattooing.

**Supplementary Information:**

The online version contains supplementary material available at 10.1038/s41598-026-42987-x.

## Introduction

Tattoos have long served social and psychological functions, including signalling status, affiliation, and beliefs, as well as supporting personal narration and aesthetic expression. In many Western contexts, their social meaning has shifted over the past century from cues associated with deviance or psychopathology to a widely accepted form of body modification^1^. Early clinical and forensic literature sometimes treated tattoos as potential indicators of psychiatric disturbance and as cues for further assessment^1,2^. As tattooing became more prevalent—and sex differences narrowed or reversed in some settings—research moved to community samples to test whether replicable psychological correlates of tattooing exist and, if so, whether they reflect maladaptive traits or normative individual differences tied to self‑expression.

Findings on personality correlates remain mixed. Several studies in non‑clinical populations report higher sensation seeking, impulsivity, and need for uniqueness among tattooed individuals, together with elevations in extraversion and openness and lower conscientiousness and agreeableness^3–6^. Associations between tattooing and risk behaviours, including substance use and unprotected sexual activity, have also been noted^7,8^. Research in incarcerated or otherwise high‑risk groups frequently documents broader psychopathology and higher rates of antisocial, narcissistic, and sadistic traits, along with anxiety and depressive symptoms^1,2^. At the same time, as body art has entered the mainstream, mean differences on broad personality traits in community samples are often small or inconsistent^5^. Contextual factors moderate these associations: the communicative impact of tattoos increases with visibility, while public self‑consciousness and expectations of negative evaluation can influence both acquisition and disclosure, complicating straightforward trait–behaviour links^9,10^. Moreover, tattoos may serve adaptive functions—identity expression, commemoration, or coping—so inferences that equate tattooing with pathology risk overgeneralisation^11^.

Although tattooing has become more normalised in many Western contexts, normalisation at the population level does not imply that tattooing is psychologically uniform or unrelated to individual differences. Community studies often find that mean differences in broad normative traits are modest and heterogeneous^5,6^, yet associations with impulsivity-related constructs and risk behaviours have also been reported^7,8^. Accordingly, the present study does not treat tattooing as a proxy for disorder. Instead, it tests whether individual differences in tattoo extent (percentage of body surface area tattooed, tBSA) are associated with dysfunctionally keyed trait expressions that are theoretically proximal to externalising behaviour and antagonistic interpersonal style, while avoiding diagnostic inference.

A notable limitation of earlier work is its reliance on normative-range trait measures (often summarised at the domain level) together with relatively coarse operationalisations of tattooing (for example, presence/absence or counts). In community samples, these approaches typically yield small to modest and sometimes inconsistent group differences^5,6^, and they may be less sensitive to associations involving dysfunctionally keyed externalising and antagonistic tendencies. The Alternative Model for Personality Disorders (AMPD) in DSM‑5 offers a dimensional framework that characterises personality pathology through five broad domains (Negative Affectivity, Detachment, Antagonism, Disinhibition, and Psychoticism), each comprising multiple facets in the full AMPD trait model^12–14^. These domains map approximately onto maladaptive poles of normative traits (e.g., Negative Affectivity with neuroticism; Detachment with low extraversion; Antagonism with low agreeableness; Disinhibition with low conscientiousness; Psychoticism with openness) but target variance at the dysfunctional end of the continua^15^. Although AMPD is organised into five broad domains, like the Five-Factor Model, it is designed to index maladaptive expressions and dysfunctionally elevated trait levels, rather than normative-range variation. In particular, impulsivity and irresponsibility within Disinhibition and hostility and callousness within Antagonism have been linked to risk‑taking, externalising behaviours, and antagonistic interpersonal styles^15,16^.

Adopting a dimensional model offers several advantages for studying tattooing in community cohorts. First, it improves construct precision by focusing on maladaptive processes—such as impulsivity and emotion dysregulation—that cut across diagnoses and predict a wide range of outcomes^5,12,13,17,18^. Second, it addresses limitations of categorical diagnoses, including heterogeneity and comorbidity, by indexing the severity and configuration of traits rather than forcing individuals into discrete categories^17,19^. Third, it aligns with contemporary views that trait continua underpin both normative and pathological personality, allowing researchers to test whether tattooing relates to specific domains without presuming clinical disorder^12,17,20^. This framework has practical value as well: by delineating trait profiles, it can clarify when observed links reflect maladaptive tendencies versus normative self‑expression^2,12^.

Measurement of tattooing itself warrants comparable refinement. Many studies rely on binary status (tattooed vs. non‑tattooed), simple counts^2,3^, or simple counts on different body parts^4,5^. These indicators can conflate different psychological and social meanings: a single large, highly visible tattoo may differ markedly from several small, concealed tattoos. Expressing tattooing as the percentage of body surface area tattooed (tBSA) provides a more sensitive and continuous index of engagement with body art, personal investment, and potential social signalling. To that end, we developed and used a Tattoo Coverage Tool (TCT) that quantifies tBSA from front and back body maps, alongside self‑reported tattoo counts. The tBSA metric is intended to capture graded variation that binary or count measures may miss and to support tests of linear associations between maladaptive traits and the extent of tattooing. This approach aligns with calls to improve phenotypic measurement in individual‑differences research, especially where behaviours vary along continua.

For personality assessment, we employed the brief form of the Personality Inventory for DSM‑5 (PID-5-BF), an open‑access instrument aligned with the AMPD domains and designed to index dysfunctionally elevated trait ranges^13^. The PID-5-BF shows robust correspondence with the full PID-5 across domains and offers efficient assessment in community settings. Its focus on maladaptive variance differentiates it from standard Five‑Factor Model measures, which target normative ranges^15,16^. Accordingly, we use the PID-5-BF here as a theoretically targeted complement to normative-range trait approaches, rather than implying that Big Five measures are uninformative for tattooing research. Prior work illustrates the value of AMPD‑aligned instruments in capturing both grandiose and vulnerable expressions of narcissism and in parsing facets of impulsivity relevant to externalising outcomes^16,21^. Applying the PID-5-BF in a non‑clinical sample enables an empirical test of whether tattoo presence and, crucially, tBSA associate with elevations in specific maladaptive domains, without inferring diagnoses.

The present study extends the literature in three complementary ways. First, we examine whether individuals with tattoos differ from those without on AMPD domains central to theories of risk and antagonism. Based on prior evidence linking tattooing with impulsivity and antisocial tendencies—particularly in higher‑risk samples—we hypothesised higher Disinhibition and Antagonism among tattooed participants, and we anticipated possible elevations in Negative Affectivity given reports of emotional instability and distress in some contexts^1,2,4,5,7^. Findings for Detachment and Psychoticism have been inconsistent in community samples, so we treated analyses of these domains as exploratory^2,5^. Second, moving beyond binary status and counts, we tested linear associations between AMPD domains and tBSA using the TCT, reasoning that Disinhibition and Antagonism would show the strongest positive relations with the percentage of body surface area tattooed. Third, we implemented this design in a non‑clinical adult sample from Cyprus, where tattooing reflects broader international trends toward normalisation. This context allows evaluation of maladaptive trait links under conditions where overt stigma is reduced, addressing the possibility that earlier associations were inflated by selection into clinical or forensic samples.

Our approach is grounded in a dimensional conceptualisation of personality pathology that emphasises transdiagnostic traits and avoids categorical inferences. By focusing on domains and facets—especially impulsivity within Disinhibition and hostility within Antagonism—we aim to test whether specific maladaptive tendencies are associated with tattooing in the general population, while recognising that tattoos can serve adaptive, identity‑related functions^11,15,16^. This design addresses two common sources of ambiguity in prior work: imprecise measurement of tattooing and reliance on broad personality constructs that may mask clinically relevant variance. In line with recommendations for trait‑based assessment, we also consider potential confounding by demographic factors and report effect sizes to contextualise any observed differences^12,14^.

In summary, the study tests two sets of hypotheses: first, compared with individuals without tattoos, those with tattoos will exhibit higher scores on Disinhibition and Antagonism, with Negative Affectivity examined as a further a priori domain and Detachment and Psychoticism assessed exploratorily; and second, greater tBSA will be positively associated with Disinhibition and Antagonism, over and above sociodemographic covariates. By integrating an AMPD‑aligned assessment (PID-5-BF) with a continuous tBSA metric (TCT), this work aims to advance the precision of psychological characterisations associated with tattooing, to reconcile mixed findings in the literature, and to distinguish maladaptive tendencies from normative self‑expression^5,6,9,10,12,15,16,21^. Analyses of Detachment and Psychoticism were treated as exploratory, and corresponding p-values are interpreted cautiously.

## Method

### Participants

A total of 280 adults, both tattooed and non-tattooed, were recruited. The target sample size was determined a priori using G*Power 3.1^22^ with power set at 0.95, a significance level of 0.001, and an anticipated effect size of 0.10. Inclusion criteria were: age 18–64 years, proficiency in Greek, and capacity to provide informed consent. No exclusion criteria were applied. The sample comprised 135 women (48.2%) and 145 men (51.8%). Regarding marital status, 202 participants (72%) were single and 48 (17%) were married; the remaining 11% were divorced or widowed. Educational attainment was as follows: 117 (41.8%) undergraduate, 72 (25.7%) postgraduate, 34 (12.1%) some university, and 54 (19.3%) secondary education. Ages ranged from 18 to 64 years (*M* = 28.0, SD = 9.5). Socioeconomic status was self-rated on a 10-point Likert scale from 1 (lowest) to 10 (highest); observed scores ranged from 3 to 10 (M = 6.25, SD = 1.40).

### Procedure

Participants were recruited from university facilities and local cafés. Researchers explained the study, answered questions, and obtained written informed consent. Consenting participants then completed a self-administered questionnaire battery assessing personality, tattoo characteristics, and demographics. During administration, no participants reported that the body maps were insufficient for indicating tattoo locations.

Participation was voluntary, without incentives, and participants could withdraw at any time. Confidentiality was maintained throughout. Ethical approval was granted by the University of Nicosia Social Sciences Ethics Review Board of the University of Nicosia (SSERB 00232).

### Measures

Demographics. Participants reported age, sex, marital status, educational attainment, and socioeconomic status.

Personality. Maladaptive personality traits were assessed using the Personality Inventory for DSM-5–Brief Form–Adult (PID-5-BF)^13^. The PID-5-BF comprises 25 self-report items yielding five domain scores—Negative Affectivity, Detachment, Antagonism, Disinhibition, and Psychoticism—each assessed with five items rated from 0 (very false or often false) to 3 (very true or often true). Domain scores range from 0 to 15 and are summed to produce a total score ranging from 0 to 75, with higher values indicating greater maladaptive trait expression. Convergent correspondence between the PID-5-BF and the full PID-5 is robust (rs ≈ 0.81–0.90)^23,24^. The instrument was translated into Greek using forward–backward translation. Three bilingual clinical psychologists were involved in the forward–backward translation process, two of whom are authors of the current study. Initially, the original items in English were translated into Greek by one of the bilingual clinical psychologists. The translated Greek version was provided to a second bilingual clinical psychologist who had no experience with the PID-5 or PID-5-BF. The original and back-translated items were reviewed and compared by the third team member, ensuring that the back-translated items addressed the same construct in content and meaning. Two minor discrepancies were identified during this stage regarding Greek cultural nuances in language use, which were addressed by all three members at a separate joint meeting. Internal consistency in the present sample was α = 0.86 for the total score; domain alphas were 0.69 (Negative Affectivity), 0.60 (Detachment), 0.68 (Antagonism), 0.74 (Disinhibition), and 0.72 (Psychoticism).

Tattoo characteristics. Tattooing was assessed using a study-developed Tattoo Coverage Tool (TCT; Appendix 1), a grid-based body-map measure designed to yield a continuous estimate of tattoo coverage that is more sensitive than binary status or simple counts in community samples. Tattoo status (tattooed vs. non-tattooed) was coded from the body maps as tBSA > 0 versus tBSA = 0. Participants reported their tattoo count and shaded tattooed regions on front and back body maps overlaid with a 1,099-square grid; men and women completed the same unisex maps. Percentage of body surface area tattooed (tBSA) was scored by counting squares shaded at least 50% as tattooed. When tattoos spanned multiple squares leaving several squares partially shaded, the shaded portions were summed during scoring to estimate the equivalent number of additional whole squares, which was added to the total. tBSA was then computed as the total number of tattooed squares divided by 1,099 and multiplied by 100. Tattoo count and tBSA were analysed separately. Formal psychometric evaluation of the TCT (interrater reliability, test-retest stability, and convergent validity with alternative coverage metrics) remains a priority for future work.

## Results

Of the 280 participants, 164 (58.6%) reported having at least one tattoo and 116 (41.4%) reported no tattoos. Six participants did not report their number of tattoos, although they did complete the TCT and provided information on tattoo coverage (percentage of body surface area tattooed, tBSA). These six participants were excluded from analyses involving tattoo count, but retained in analyses involving tBSA and tattoo status. Descriptive statistics for tattoo count, percentage of body surface area tattooed (tBSA), PID-5-BF total score, and the five domains are presented in Table 1. The intercorrelation matrix for tattoo variables, PID-5-BF scores, age, and socioeconomic status is presented in Table 2. Sex was examined separately using t tests (Table 3).

**Table 1:** Descriptive statistics of the variables of interest in the study.

	*N*	Minimum	Maximum	Range	M	SD
Age	278	18	64	46	28.00	9.495
Socioeconomic status	248	3	10	7	6.25	1.403
Number of tattoos	274	0	35	35	2.75	4.86
Tattoo body coverage %	279	0	34.64	34.64	2.08	4.29
Negative affectivity	278	0	15	15	6.62	3.37
Detachment	277	0	12	12	3.69	2.72
Antagonism	280	0	12	12	2.92	2.79
Disinhibition	279	0	15	15	5.21	3.28
Psychoticism	278	0	14	14	4.25	3.10
PID-5-BF total score	272	2	62	60	22.78	10.88

**Table 2:** Correlation matrix of tattoo variables, PID-5-BF scores, age, and socioeconomic status.

	1	2	3	4	5	6	7	8	9	10
1. Age	–									
2. Socioeconomic status	− 0.02	–								
3. Number of tattoos	0.01	0.03	–							
4. tBSA	0.04	0.06	0.78^**^	–						
5. Negative affectivity	− 0.05	0.01	− 0.01	− 0.11	–					
6. Detachment	0.06	0.08	0.01	0.07	0.26^**^	–				
7. Antagonism	− 0.17^**^	0.20^**^	0.10	0.26^**^	0.25^**^	0.38^**^	–			
8. Disinhibition	− 0.17^**^	0.16^*^	0.14^*^	0.21^**^	0.24^**^	0.35^**^	0.49^**^	–		
9. Psychoticism	− 0.20^**^	0.14^*^	0.13^*^	0.11	0.39^**^	0.42^**^	0.48^**^	0.52^**^	–	
10. PID-5-BF total	− 0.15^*^	0.18^**^	0.10	0.16^**^	0.62^**^	0.66^**^	0.73^**^	0.74^**^	0.79^**^	–

**Correlation is significant at the 0.01 level (2-tailed).

*Correlation is significant at the 0.05 level (2-tailed).

**Table 3:** Independent samples t-test on the number of tattoos, tattoo body coverage, the five personality domains, and the PID-5-BF total score by sex.

		*N*	*M*	*SD*	*t*	*df*	*p*	*95%CI [LL*,* UL]*	*d*
Number of tattoos	Female	131	2.65	4.36	− 0.32	272	0.75	[− 1.35, 0.97]	− 0.04
	Male	143	2.84	5.30					
Tattoo body Coverage %	Female	134	1.16	2.08	− 3.62	187.34	0.01	[− 2.74, − 0.81]	− 0.42
	Male	145	2.94	5.49					
Negative affectivity	Female	134	7.29	3.45	3.28	276	0.01	[0.52, 2.09]	0.40
	Male	144	5.99	3.19					
Detachment	Female	134	3.24	2.51	− 2.72	275	0.01	[− 1.52, − 0.24]	− 0.33
	Male	143	4.12	2.84					
Antagonism	Female	135	2.26	2.29	− 4.39	260.97	0.01	[− 2.1, − 0.8]	− 0.52
	Male	145	3.71	3.20					
Disinhibition	Female	134	4.29	2.80	− 4.69	272.09	0.01	[− 2.51, − 1.02]	− 0.56
	Male	145	6.06	3.47					
Psychoticism	Female	133	3.65	2.92	− 3.19	276	0.01	[− 1.89, − 0.45]	− 0.38
	Male	145	4.81	3.16					
PID-5-BF	Female	130	20.79	9.80	− 3.08	268.64	0.01	[− 6.53, − 1.44]	− 0.37
	Male	142	24.77	11.50					

Sex differences were examined using independent samples t tests for tattoo count, tBSA, the five AMPD domains, and the PID-5-BF total. There was no significant sex difference in tattoo count. Women scored higher on Negative Affectivity, whereas men scored higher on tBSA, Antagonism, Detachment, Disinhibition, Psychoticism, and the PID-5-BF total (Table 3).

Group differences by tattoo status (yes/no) were tested using independent samples t tests. There were no significant differences between tattooed and non-tattooed participants in Detachment, Negative Affectivity, or Antagonism. In contrast, tattooed participants scored higher on Disinhibition (*M* = 5.84, *SD* = 3.3) than non-tattooed participants (*M* = 4.33, *SD* = 3.07), 95% *CI* [0.73, 2.28], *t* (271) = 3.83, *p*<.01, *d*= 0.47. Tattooed participants also had higher PID-5-BF total scores (*M* = 24.21, *SD* = 10.98) than non-tattooed participants (*M* = 21.05, *SD* = 10.59), 95% *CI* [ 0.53, 5.8] *t* (266) = 2.37, *p*=.02, *d*= 0.29.

Associations between tattooing and maladaptive traits were examined using Pearson correlations. tBSA correlated positively with Antagonism (*r* = .26, *p* < .01), Disinhibition (*r* = .21, *p* < .01), and the PID-5-BF total (*r* = .16, *p* = .01). tBSA was not significantly associated with Detachment or Negative Affectivity. When the tattoo count was used instead of tBSA, smaller associations were observed with Disinhibition (*r* = .14, *p* < .05) and Psychoticism (*r* = .13, *p* < .05), suggesting that tBSA is a more sensitive indicator of the extent of tattooing.

Regression models were used to assess whether maladaptive traits predict tBSA. In separate models, Antagonism significantly predicted tBSA, accounting for 6.8% of the variance, *F*(1,277) = 20.21, *β* = 0.26, *t* = 4.49, *p*<.001, and Disinhibition accounted for 4.6% of the variance, *F*(1,276) = 13.23, *β* = 0.21, *t* = 3.64, *p*< .001. Entered jointly, Antagonism and Disinhibition explained 7.7% of the variance in tBSA, *F*(2,275) = 11.48, *p*< .001; Antagonism remained a significant predictor (*b* = 0.20, *t* = 3.55, *p*< .01), whereas Disinhibition attenuated to non-significance (*b* = 0.11, *t* = 1.68, *p*= .09).

A hierarchical multiple regression examined whether Antagonism and Disinhibition predicted tBSA after controlling for sex, age, and socioeconomic status (Step 1). Step 1 explained approximately 3.8% of the variance in tBSA (R² = 0.038, *p* < .05; *F*(3, 240) = 3.18, *p*<.05). The addition of Antagonism and Disinhibition in Step 2 explained an additional approximately 5.2% of variance (ΔR² = 0.052, *F*(2, 238) = 6.81, *p* < .001), with the final model explaining approximately 9.0% of the variance (R^2^= 0.09, *F*(5, 238) = 4.73, *p*< .001). In the final model, Antagonism was a significant positive predictor (β = 0.23, *p* = .003), whereas Disinhibition was not (β = 0.04, *p* = .60). Full results are presented in Table 4.

**Table 4:** Hierarchical multiple regression analyses of disinhibition and antagonism on tattoo body coverage, controlling for age, sex, and socioeconomic status.

		B	β	t	*p*
Step 1	*R*^2^= 0.04, *F*(3, 240) = 3.18, *p<*.05				
	Sex	0.08	0.02	0.26	0.80
	Age	17.27	0.18	2.90	0.004
	Socioeconomic status	2.49	0.08	1.18	0.24
Step 2	*ΔR*^2^= 0.05, *F*(2, 238) = 6.81, *p*< .001				
	Sex	0.33	0.07	1.08	0.28
	Age	10.30	0.11	1.67	0.10
	Socioeconomic status	0.56	0.02	0.26	0.79
	Antagonism	3.63	0.23	3.01	0.003
	Disinhibition	0.57	0.04	0.53	0.60

*R*^2^= 0.09, *F*(5, 238) = 4.73, *p*< .001.

We also examined whether the Antagonism effect would remain significant when all five PID-5-BF domains were added at Step 2. In this analysis, Step 1 (age, sex, and socioeconomic status) explained 4.0% of the variance in tBSA (R² = 0.04, *p* < .05). Step 2, which added all five PID-5-BF domains, explained an additional 8.0% of the variance (ΔR² = 0.08, *p* < .001), with the full model explaining 12.0% (R² = 0.12). Antagonism remained significant and showed the strongest effect (β = 0.29, *p* < .001). Negative Affectivity also made a small negative unique contribution to the explained variance in tBSA (β = − 0.18, *p* = .02). Results are presented in Table 5.

**Table 5:** Hierarchical multiple regression analyses of the five domains of the PID-5-BF on tattoo body coverage (tBSA), controlling for age, sex, and socioeconomic status.

		B	β	t	*p*
Step 1	*R*^2^= 0.04, *F*(3, 234) = 3.12, *p<*.05				
	Sex	0.06	0.01	0.21	0.83
	Age	16.77	0.18	2.83	0.005
	Socioeconomic status	2.72	0.08	1.3	0.19
Step 2	Δ*R*^2^ = 0.08; Δ*F*(5, 229) = 4.37, *p* < .001				
	Sex	0.39	0.09	1.29	0.19
	Age	3.87	0.04	0.59	0.56
	Socioeconomic status	− 0.02	− 0.001	− 0.01	0.99
	Negative affectivity	-2.38	− 0.18	-2.38	0.02
	Detachment	− 0.67	− 0.04	− 0.55	0.58
	Psychoticism	0.24	0.02	0.19	0.85
	Antagonism	4.54	0.29	3.57	< 0.001
	Disinhibition	1.17	0.08	1.04	0.29

*R*^*2*^*=* 0.12, *F*(8, 229) = 3.99, *p*< .001.

## Discussion

This study examined associations between maladaptive personality traits and tattooing in a community sample. DSM-5 Alternative Model for Personality Disorders (AMPD) domains were assessed with the Personality Inventory for DSM-5–Brief Form (PID-5-BF), and tattooing was quantified as the percentage of body surface area tattooed (tBSA). In this protocol, tBSA was computed with the Tattoo Coverage Tool (TCT), which converts shaded regions on front and back body maps into a percentage of total grid area. Three findings emerged. First, individuals with tattoos scored higher than those without on Disinhibition and on the PID-5-BF total score, whereas Antagonism, Negative Affectivity, Detachment, and Psychoticism did not differ by tattoo status. Second, tBSA showed graded positive associations with Antagonism, with Disinhibition, and with the overall maladaptive trait load, whereas simple tattoo counts showed only weaker associations. In multivariable models, Antagonism remained the only independent predictor of tBSA after adjustment for Disinhibition and demographic covariates. Third, men reported higher tBSA and higher scores on Antagonism, Detachment, Disinhibition, Psychoticism, and the PID-5-BF total score, while women scored higher on Negative Affectivity. The magnitudes of the main effects were small to moderate (e.g., d ≈ 0.47 for Disinhibition; d ≈ 0.29 for the total score), consistent with contemporary community-based research and insufficient to support diagnostic inference.

These findings clarify an uneven literature by focusing on maladaptive trait ranges and by improving the measurement of tattoo exposure. Community-based studies grounded in broad trait models generally report that tattooed individuals show higher sensation seeking, impulsivity, and need for uniqueness, alongside lower agreeableness and conscientiousness and higher extraversion and openness^3–6^. Effect sizes are typically modest and heterogeneous across samples, likely reflecting the normalisation of tattooing in many societies^5^. Our results align with this pattern for impulsivity-related variance: tattooed participants scored higher on Disinhibition, consistent with links between tattooing and risk behaviours such as substance use and unprotected sex^7,8^. By adopting the DSM-5 Alternative Model for Personality Disorders, which captures dysfunctionally elevated variance in domains including impulsivity/irresponsibility (Disinhibition) and hostility/callousness (Antagonism), the study provides greater specificity than Five-Factor Model indices^12–16^. The absence of between-group differences in Antagonism suggests that antagonistic traits do not differentiate tattooed from non-tattooed individuals when tattooing is dichotomised, a pattern compatible with the contemporary normalisation of tattooing and in contrast to earlier clinical and forensic reports of broader psychopathology among tattooed samples^1,2^. Moreover, indexing tattooing by percentage of body surface area (tBSA) using the Tattoo Coverage Tool, rather than by simple counts, offers a more sensitive exposure metric that helps to resolve graded associations between tattoo extent and antagonistic and disinhibitory traits.

Expressing tattooing as a percentage of body surface area (tBSA) strengthens the measurement. tBSA captured variance that simple counts attenuated and showed clear positive associations with Antagonism and Disinhibition. This pattern supports the view that the extent of tattooed skin coverage is a more sensitive indicator of engagement than a tally of pieces, although the degree to which coverage is visible depends on placement and everyday clothing. Prior work indicates that the communicative impact of tattoos scales with visibility and extent, and that public self-consciousness and anticipated evaluation influence acquisition and disclosure^9,10^. In the present regressions, Antagonism accounted for unique variance in tBSA after adjustment for Disinhibition and demographic covariates. A plausible interpretation, which should be treated as tentative, is that disinhibitory tendencies are most salient during initiation and opportunistic acquisition, whereas antagonistic interpersonal style relates more closely to cumulative investment in larger, more prominent, or assertive designs once variance shared with impulsivity is controlled. Longitudinal data are needed to test these temporal hypotheses.

Tattoo visibility and placement likely moderate the social signalling value of tattoos and may therefore shape associations with personality. Although the body maps used in the Tattoo Coverage Tool contain location information, the present scoring approach was intentionally limited to an overall, continuous coverage index (tBSA). We did not derive region-specific or visibility-weighted indices in the present manuscript because such indices require a priori region definitions and weighting rules (and ideally validation) to avoid post hoc analytic flexibility, and because strongly visible placements (for example, face/neck/hands) were very rare in this community sample, limiting statistical power and the stability of estimates. Future research with larger samples could preregister region-based scoring (for example, visible tBSA versus typically covered tBSA, or a visibility-weighted tBSA) and incorporate contextual measures that determine everyday visibility (for example, usual clothing), which may be central to anticipated evaluation and public self-consciousness^9,10^. In addition, the present study did not assess tattoo painfulness or pain experienced during tattooing; collecting pain ratings and placement-specific “cost” indicators would enable tests of whether higher-cost or rarer placements show different trait associations.

The null findings for Detachment and Negative Affectivity, observed in both binary comparisons and with respect to tBSA, warrant comment. Detachment indexes the maladaptive counterpart of extraversion and positive affect (e.g., social withdrawal, anhedonia). Reports of higher extraversion among tattooed individuals likely reflect normative trait variance and thus fall outside the construct range emphasised by the PID-5-BF, which targets dysfunctionally elevated traits^4,5,15^. At the bivariate level, tattooing and tBSA were not associated with Negative Affectivity in this sample, suggesting that tattooing was not linked to broadly elevated negative emotionality. However, when all five PID-5-BF domains were entered simultaneously (Table 5), Negative Affectivity showed a small independent association with tBSA in the negative direction (β = − 0.18, *p* = .02), alongside Antagonism (β = 0.29, *p* < .001). Given the absence of a bivariate association between tBSA and Negative Affectivity, this pattern may reflect shared variance among correlated trait domains (a suppression effect) rather than a straightforward zero-order relation. Accordingly, we interpret the Negative Affectivity coefficient cautiously and treat it as hypothesis-generating pending preregistered replication. Tattoos may serve heterogeneous functions (for example, identity expression or commemoration^11^. However, the present data do not directly test whether tattooing is adaptive. Psychoticism, which captures maladaptive aspects of openness (e.g., eccentricity, perceptual dysregulation), showed a weak association with tattoo counts but not with tBSA, suggesting that openness-related variance may influence the decision to acquire tattoos yet does not scale with the proportion of body surface area tattooed. Given the relatively small effect sizes, additional research is required before firm conclusions can be drawn regarding Psychoticism.

Interpretation should consider broader social trends in the normalisation of tattooing. Tattoo prevalence in this sample was high, and sex differences were observed in both personality traits and tBSA. Earlier forensic and clinical research, often conducted in settings where tattoos function as markers of group affiliation or deviance, reported broader psychopathology and higher antisocial and narcissistic traits among tattooed individuals^1,2^. By contrast, community studies from contexts where tattooing is common typically show modest, domain-specific differences^5^. The present findings align with this latter pattern: tattoo presence was associated with higher Disinhibition and a small increase in overall maladaptive trait load, while tBSA showed graded associations with Antagonism and Disinhibition. Collectively, the evidence indicates that tattooing per se is not a proxy for psychopathology in the general population, whereas higher percentages of body surface area tattooed relate modestly to antagonistic and disinhibited tendencies.

Sex differences mirrored established personality patterns. Men scored higher on Antagonism and Disinhibition and reported greater tBSA, whereas women scored higher on Negative Affectivity, consistent with broader externalising versus internalising distinctions^15,16^. Gendered norms regarding visibility, placement, and style may contribute to differences in tBSA and to differential social feedback, which may, in turn, influence subsequent acquisition^9,10^. Whether sex moderates associations between specific traits and tBSA remains unresolved; adequately powered, pre-registered tests of interaction effects and mediation via motivations (identity expression, memorialisation, aesthetic preference) would be informative^2,12,21^.

Methodologically, two features strengthen the contribution. First, the use of the PID-5-BF situates interpretation within the DSM-5 AMPD, which prioritises transdiagnostic trait dimensions relevant to externalising and interpersonal functioning and avoids key limitations of categorical diagnoses, including heterogeneity, comorbidity, and threshold effects^12–14,17,19,20^. Second, quantifying tattooing as the percentage of body surface area (tBSA) provides a continuous exposure metric that is more sensitive than binary status or simple counts. This operationalisation supports linear and dose-response tests and differentiates the psychological implications of a single large, visible piece from those of multiple small, concealed tattoos that may share a count yet differ substantially in tBSA.

Model explanatory power was modest. After adjustment for age, sex, and socioeconomic status, Antagonism and Disinhibition together accounted for an additional 5.2% of variance in tBSA (ΔR² = 0.052), and the full model explained 9.0% (R² = 0.090). These values are typical for personality–behaviour associations in community samples and indicate that many unmeasured influences (cultural norms, peer networks, identity processes, aesthetic preferences, and opportunity structures) likely contribute to the proportion of body surface area tattooed^4,6,9,10^. Accordingly, the findings should be understood as group-level tendencies with limited predictive utility for individuals.

### Limitations

Several limitations qualify interpretation. The cross-sectional design precludes causal inference; it remains unclear whether elevations in Disinhibition and Antagonism predispose individuals to greater tBSA or whether experiences associated with more visible tattooing shape trait expression over time. The convenience sample, recruited from university facilities and local venues and skewing young, may limit generalisability to older or more heterogeneous populations. Reliance on self-report for all variables raises concerns about common method variance and social desirability; future work should incorporate behavioural indices of impulsivity and antagonism and informant reports, or at the very least, include measures of socially desirable responding.

Using additional modalities could change the magnitude, and potentially the pattern, of associations observed here. Associations may attenuate when predictors and tattoo exposure are measured using different sources (for example, informant reports or behavioural tasks), because shared-method variance and socially desirable responding are reduced. At the same time, some links could strengthen if self-report underestimates socially undesirable tendencies. Informant reports may be particularly informative for antagonism-related tendencies that are expressed in observable interpersonal behaviour, while behavioural measures of impulsivity may index components of self-control that are only partly captured by self-report Disinhibition. Multi-method designs (for example, combining self-report, informant report, and behavioural indices, and where feasible using independently coded tattoo coverage) would therefore provide stronger tests of whether antagonistic and disinhibited tendencies are robustly associated with tBSA.

The Greek PID-5-BF demonstrated good internal consistency for the total score but only moderate reliability for some domains, which may attenuate domain-level associations; tests of measurement invariance by sex and age within the Greek context would strengthen confidence in comparisons. With respect to tattoo measurement, tBSA quantifies the proportion of body surface area tattooed but does not capture visibility, placement, salience, or stylistic features that may moderate social signalling and trait associations^9,10^. Moreover, because highly visible placements were uncommon and the sample size was modest, any post hoc location-stratified analyses would likely be underpowered and yield unstable estimates; these questions are better addressed in larger studies with preregistered region definitions and weighting rules. The psychometric properties of the Tattoo Coverage Tool warrant formal evaluation, including inter-rater reliability, test–retest stability, and convergent validity with alternative coverage metrics. Tattoo counts were self-reported without a standardised definition of a discrete tattoo, introducing additional measurement error.

Unmeasured covariates, such as motivations for tattooing, peer and partner influences, risk behaviours, and psychiatric symptoms, may partially account for associations with Disinhibition and Antagonism^4,6–8^. Cultural specificity also matters: the sample was drawn from Cyprus, and norms around tattooing and visibility vary across societies and subcultures; replication in other contexts is needed. Although several statistical tests are reported, the principal inferences were based on a small set of a priori hypotheses focused on Disinhibition and Antagonism (and Negative Affectivity specified a priori), while analyses of Detachment and Psychoticism were exploratory. We did not apply a blanket multiplicity correction across all tests because strict familywise-error controls can be overly conservative when outcomes are correlated, increasing Type II error in community samples with modest effects. Accordingly, we report exact p-values and effect sizes throughout and interpret exploratory findings as hypothesis-generating pending preregistered replication. Future studies could administer both PID-5 and a Big Five instrument (ideally with facet scales) to test whether tattooing is better explained by normative trait variation, maladaptive trait elevation, or a combination, and whether tBSA relates differently across these measurement levels.

## Conclusions

In a community sample with high tattoo prevalence, tattoo presence was associated with higher Disinhibition and a small increase in overall maladaptive trait load, whereas other AMPD domains did not differ by status. Quantifying tattooing as the percentage of body surface area tattooed (tBSA) was more informative than simple counts, revealing graded associations of tBSA with Antagonism and Disinhibition and an independent association with Antagonism after adjusting for Disinhibition and demographic covariates. Effect sizes were small to moderate and do not warrant diagnostic inference. These findings support AMPD-aligned trait assessment and the use of tBSA as an exposure metric in future research. Longitudinal, multi-method studies that incorporate placement and visibility, motivations, and social context, alongside formal validation of tBSA measures, are needed to test whether antagonistic and disinhibited tendencies prospectively track increases in tattooed body surface area and whether tBSA offers incremental predictive value beyond binary status and counts for behavioural and psychosocial outcomes.

## Supplementary Information

Below is the link to the electronic supplementary material.

Supplementary Material 1

## Data Availability

The datasets used and analysed during the current study are available from the corresponding author on reasonable request.
