# Supplementary figures and images for: Associations between tattooed body surface area and maladaptive personality traits in a community sample
Source: Sci Rep. 2026 Mar 8;16:8642. doi: 10.1038/s41598-026-42987-x (PMC12979667; doi:10.1038/s41598-026-42987-x)

**Appendix**


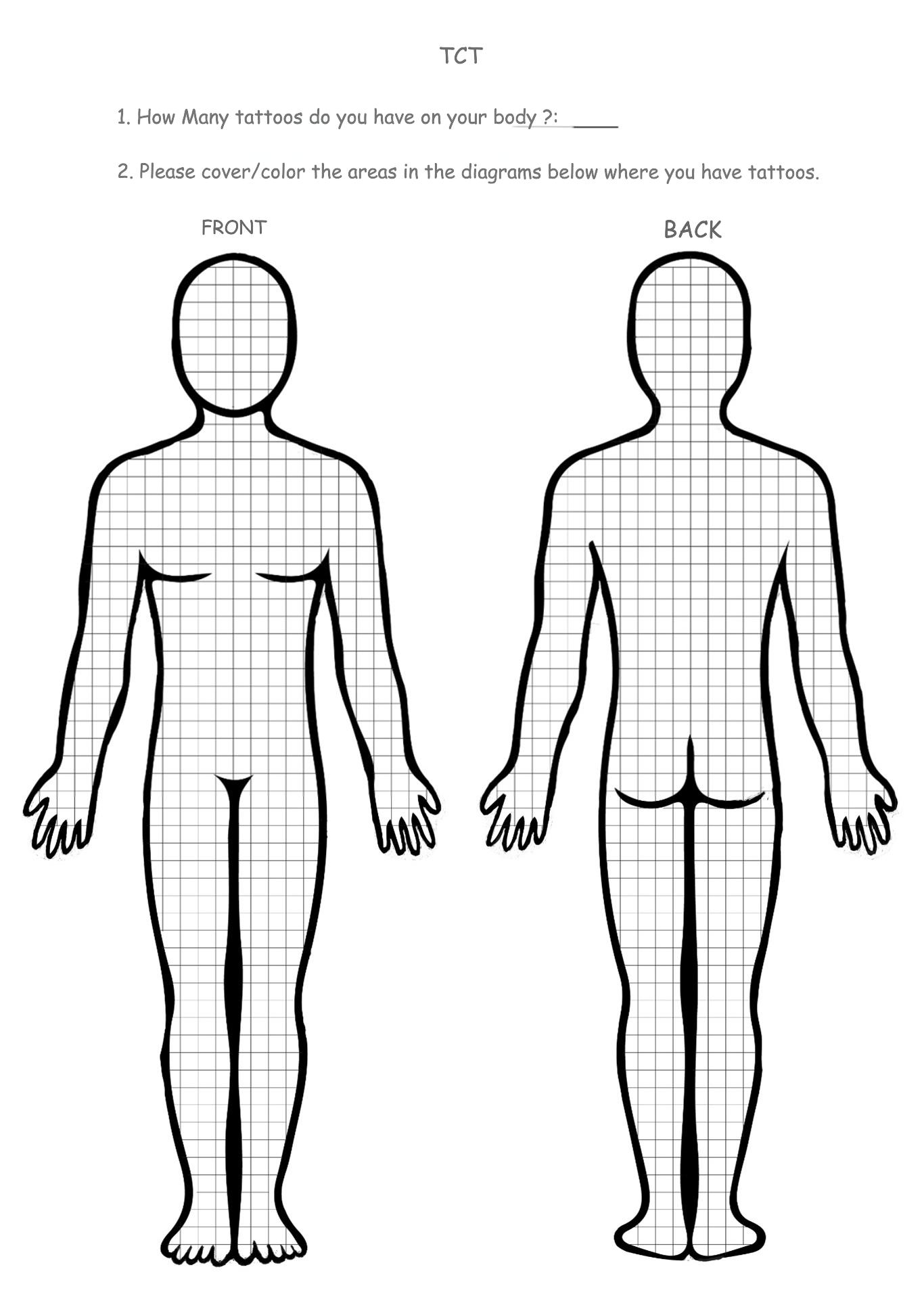
Tattoo Coverage Tool

Supplement: Supplementary file 1 — Supplementary Material 1 [file 41598_2026_42987_MOESM1_ESM.docx]
